# *Circaea mollis* Siebold & Zucc. Alleviates postmenopausal osteoporosis in a mouse model via the BMP-2/4/Runx2 pathway

**DOI:** 10.1186/s12906-020-02914-7

**Published:** 2020-04-22

**Authors:** Ji Hye Park, Yang Ju Son, Chang Ho Lee, Chu Won Nho, Gyhye Yoo

**Affiliations:** 1 Smart Farm Research Center, Gangneung Institute of Natural Products, Natural Products Research Center, Korea Institute of Science and Technology (KIST), Gangneung, Gangwon-do 25451 South Korea; 2 College of Biology, Gangneung-Wonju National University, Gangneung, 25457 South Korea

**Keywords:** *Circaea mollis* Siebold & Zucc, Postmenopausal osteoporosis, Osteoblast differentiation, Runt-related transcription factor 2, Bone morphogenetic protein 2/4

## Abstract

**Background:**

*Circaea mollis* Sieb. & Zucc. has been used as a traditional herbal medicine in Hani Ethnopharmacy and possesses anti-arthritic activities. This study aimed to investigate the effect of *Circaea mollis* Siebold & Zucc on postmenopausal osteoporosis.

**Methods:**

For in vitro study, MCF7 breast cancer cells and MC3T3-E1 pre-osteoblast cells were utilized to estimate estrogenic and osteogenic activity. Osteoblastic markers were measured by western blot and real-time PCR. For in vivo study, female mature C57BL/6 mice were ovariectomized and oral administrated with 10 mg/kg and 40 mg/kg of EECM respectively.

**Results:**

EtOH extract of *Circaea mollis* Siebold & Zucc. (EECM) increased alkaline phosphatase activity and osteoblast marker levels at day 7 during differentiation of mouse preosteoblasts. EECM reduced osteoclast differentiation and bone resorption in an osteoblast-osteoclast primary co-culture system. In ovariectomized mice, EECM prevented the decrease in bone mineral density and recovered OSX and Runx2 via BMP2/4, Smad1/5/9 and p38.

**Conclusions:**

The results suggest that EECM may be effective in preventing bone loss, offering a promising alternative for the nutritional management of postmenopausal osteoporosis.

## Background

Postmenopausal osteoporosis, which is caused by estrogen deficiency, constitutes the most common disease of osteoporosis and typically affects women within 10–15 years after menopause, resulting in approximately 40% of women experiencing one or more fractures after menopause [1, 2]. For the treatment of postmenopausal osteoporosis, agents including hormone replacement therapy (HRT), selective estrogen receptor modulators (SERMs), and bisphosphonates have been utilized; most of these therapeutic agents are focused on the inhibition of osteoclastogenesis. HRT is the most common method used to treat menopausal syndromes, although it is not recommended for long-term therapy owing to serious side effects, including breast cancer and cardiovascular diseases [3, 4]. In addition, for many decades, inhibition of osteoclastogenesis has been a common target for postmenopausal osteoporosis because it is known that estrogen suppresses osteoclastogenesis through modulation of RANK signaling and induction of osteoclast apoptosis [5, 6]. Although abundant suppression of osteoclastogenesis agents were developed, the efficacy in recovering bone mass is relatively less. Therefore, the development of novel medicines for treating osteoporosis without adverse side-effects is required.

Bone homeostasis is important and tightly regulated by various signaling factors and hormones. In bone remodeling, two specialized cell types, osteoblasts and osteoclasts, play a major role in rebuilding and destroying bone matrix, respectively. Osteoblasts, originated from mesenchymal stem cells (MSCs), are critical for the maturation and mineralization of bone. These cells release a variety of proteins that induce the bone formation process, including Runt-related transcription factor 2 (Runx2) and Sp7 transcription factor (also called OSX), alkaline phosphatase (ALP, an early stage osteoblast differentiation marker), osteopontin (OPN, a mineralization inhibitor), and osteoprotegerin (OPG, an inhibitor of osteoclast differentiation) [7, 8]. In turn, osteoclasts are bone-resorbing multinucleated giant cells differentiated from monocyte-macrophage lineage precursor cells. The differentiation of osteoclasts is induced by two crucial cytokines: macrophage colony-stimulating factor (M-CSF) and receptor activator of nuclear factor-κ B ligand (RANKL). The former is important for the proliferation and survival of osteoclast precursor cells, along with the constitutive expression of RANK [9]. RANKL triggers the signaling for osteoclast differentiation by binding to its receptor, RANK, on the surface of osteoclast precursor cells. Imbalance between osteoblasts and osteoclasts causes disorders of bone homeostasis and leads to skeletal dysfunctions such as osteopetrosis, renal osteodystrophy, PAGET’s disease of bone, and osteoporosis. Thus, to effect a cure for osteoporosis, it is necessary to induce a balance of the two bone cell types.

Small molecules in medicinal plants serve as a defense mechanism for the plants’ survival and have also been shown to exert beneficial effects against human diseases. In particular, several studies have shown that crude extracts from plants and their compounds [10, 11] have curable effects on osteoporosis. Considering the potential of phytoestrogens, it is critical to develop new candidates from plants for the treatment of osteoporosis. *Circaea mollis* Siebold & Zucc. constitutes a traditional herbal medicine that is used in Hani ethnopharmacy and throughout Asia. Recently, *Circaea mollis* Siebold & Zucc. has been reported to exhibit anti-arthritic activity, and galuteolin, vitexin and isoorientin were found in this plant [12]. Though *Circaea mollis* Siebold & Zucc.is a phytoestrogens-rich plant, its functional effects on osteoporosis and bone health remain to be elucidated. Therefore, the major aim of this study was to determine whether the ethanol extract of *Circaea mollis* Siebold & Zucc. (EECM) demonstrates potential as a therapeutic option for osteoporosis and ameliorating bone loss.

## Methods

### Reagents

EECM was provided by the Korea Institute Science Technology Natural Products Library (Gangneung, South Korea). Alpha minimum essential medium (α-MEM), Dulbecco’s modified Eagle’s medium, penicillin-streptomycin, and phosphate buffered saline (PBS) were purchased from Welgene (Gyeongsan, South Korea). Fetal bovine serum was purchased from Gibco, Gaithersburg, MD. Antibodies against β-actin, Runx2, OPG, OSX, BMP2/4, ALP, and secondary anti-rabbit and anti-mouse antibodies were purchased from Santa Cruz Biotechnology, Dallas, TX. Antibodies against phospho-extracellular signal-regulated protein kinase (p-ERK), ERK, and COL1A1 were purchased from Cell Signaling Technology (Danvers, MA). Alizarin Red S (ARS), 1,25-dihydroxyvitamin D3, L-ascorbic acid, β-glycerophosphate, ICI 182,780 and genistein were purchased from Sigma-Aldrich Chemical Co. (St. Louis, MO). Ultra Pure bovine serum albumin (BSA) was purchased from GenDEPOT (Katy, TX). Noggins was purchased from Peprotech (London, U.K.).

### Cell culture

Mouse pre-osteoblast MC3T3-E1 subclone 4 and mouse monocyte macrophage RAW 264.7 cells (American Type Culture Collection (ATCC), Manassas, VA) were cultured in α-MEM (without L-ascorbic acid) and MEM containing 10% fetal bovine serum and 1% penicillin streptomycin in a 5% CO_2_ incubator at 37 °C. Human breast cancer MCF-7 cells (ATCC) were cultured in MEM containing 10% fetal bovine serum and 1% penicillin streptomycin in a 5% CO_2_ incubator. For phytoestrogen stimulation, MCF-7 cells were cultured in the experimental medium, which contained phenol red-free MEM supplemented with 10% charcoal dextran-treated fetal bovine serum, 2 mmol L- 1 L-glutamine, 1 mmol L-1 sodium pyruvate, and 1% penicillin streptomycin in a 5% CO_2_ incubator.

### Luciferase reporter assays

MCF7 cells were transfected with an estrogen response element luciferase plasmid and qRL-CMV plasmid using iN-fect in vitro transfection Reagent (INtRON, Seoul, South Korea) according to the manufacturer’s instructions. All cells were allowed to reach 50–80% confluence prior to transfection. After 24 h, the medium was placed with fresh medium containing EECM (2.5–40 μg/mL) or genistein (10 μM) for 24 h. Then, cells were lysed and assayed for luciferase expression using a Dual Luciferase Assay kit (Promega, Madison, WI), according to the manufacturer’s instructions. Luciferase activity was detected using a Synergy HT multi-microplate reader (BioTek Instruments, Winooski, VT). *Renilla* luciferase activity was used to normalize transfection efficiencies.

### ALP activity measurement

MC3T3-E1 cells were seeded in a 24 well plate with 2 × 10^4^ cells/well. When cells reached confluence, osteogenic differentiation was induced by addition of 50 μg/mL L-ascorbic acid and 10 mM β-glycerophosphate. MC3T3-E1 cells were treated at confluence with EECM (2.5–20 μg/mL) in differentiation medium for 7 days. Inhibitors; Noggin (200 ng/mL) and ICI182.780 (10 μM) were treated 1 h before the treat EECM. After 3 days, cells were retreated with same treatments. After 7 days of incubation, cells were washed with PBS twice and lysed with 0.1% Triton-X-100 lysis buffer. ALP activity was measured using the SensoLyte pNPP Alkaline Phosphatase Assay Kit (AnaSpec, Fremont, CA) as directed by the manufacturer.

### Measurement of mineralization

MC3T3-E1 cells were differentiated with EECM (2–20 μg/mL) for 14 days. Cells were then washed with PBS, fixed with 4% formalin for 15 min, and stained with 2% ARS (pH 4.6). The ARS-stained cells were solubilized with 10% cetyl chloride in 10 mM sodium phosphate by shaking. The absorbance of the solubilized stain was measured at 504 nm on a Synergy HT Multi-microplate reader.

### Tartrate-resistant acid phosphatase (TRAP) activity

RAW 264.7 cells were seeded in a 24-well plate with 1 × 10^5^ cells/well. After incubation for 24 h, the medium was replaced with α-MEM containing 100 ng/mL receptor activation of RANKL (Peprotech, London, U.K.) for cell differentiation. RAW 264.7 cells were treated with EECM (2–20 μg/mL) in differentiation medium for 6 days. After 6 days of cell differentiation, the medium was removed and the cell monolayer was gently washed twice with PBS. Then the cells were lysed with 200 μL of 0.1% Triton X-100. An aliquot of cell lysate was added to TRAP solution (10 mM sodium tartrate and 0.1 M sodium acetate, pH 5.2) and p-nitrophenyl phosphate (10 mM) subsequently incubated at 37 °C. The reaction was stopped with 0.7 N NaOH and the absorbance was measured at 405 nm using a Synergy HT Multi-microplate reader.

### Western blotting

Cells were lysed in RIPA buffer (Sigma-Aldrich) containing a protease inhibitor cocktail and sonicated for protein preparation. Protein concentrations were determined using the Bradford protein assay kit with BSA as the standard. Protein samples were denatured in sodium dodecyl sulfate sample buffer and resolved on a 10% sodium dodecyl sulfate-polyacrylamide gel. These proteins were then transferred to polyvinylidene difluoride membranes. The membranes were blocked with PBS-T containing 3% BSA. Subsequently, the membranes were reacted with specific antibodies. Loading differences were normalized using an anti-β-actin antibody. Peroxidase activity on the membrane was visualized by using an ECL Western blotting detection system (Fujifilm, Tokyo, Japan).

### Quantification of gene expression by real-time reverse transcription polymerase chain reaction (RT-PCR)

Total RNAs were extracted from cells using a Total RNA Isolation Kit (GeneALL Biotechnology, Seoul, South Korea) according to the manufacturer’s instruction. cDNAs were synthesized using the PrimeScript 1st strand cDNA synthesis Kit (TaKaRa, Osaka, Japan). Quantitative real-time PCR was performed using SYBR Green working solution iTaqTM Universal SYBR Green Supermix (Bio-Rad, Hercules, CA) using a custom PCR master mix and the following conditions: 40 cycles of 95 °C for 30 s, 55 °C for 30 s, and 72 °C for 30 s. mRNA expression levels were normalized to *Gapdh* expression. The primer sequences were listed in Supplementary Table 1.

### Animals

Seven-week-old female C57BL/6 mice were purchased from Central Lab. Animal Inc. (Seoul, South Korea) and housed at the Korea Institute of Science and Technology at a constant temperature (25 ± 2 °C) with a 12 h light and 12 h dark cycle with free access to food and water. Mice were provided with regular bedding and specific pathogen free cages. Animal studies were conducted in accordance with institutional and national guidelines, and all experimental procedures were approved by the Animal Use and Care Committee of the Korea Institute of Science and Technology (2016–083), Gangneung, Korea.

### Primary cell culture

Bone marrow-osteoblast coculture was conducted according to the method described by Takahashi et al. [13] For primary cell culture of osteoblasts, calvarial bones were dissected from 7-month-old female C57BL/6 J mice. For the isolation of osteoblasts, the calvariae were washed in PBS and treated for 15 min with PBS followed by consecutive 25-min treatments with collagenase (collagenase type IA, Sigma-Aldrich). The resulting cell suspensions were spun down and re-suspended in culture medium (α-MEM, supplemented with 10% fetal calf serum (FCS, Gibco) and penicillin/streptomycin) and cell suspensions were pooled. The cells were allowed to adhere to a 100 mm culture plate that was pre-coated by collagen. After 24 h, non-adherent cells were removed and the remaining cells cultured overnight in culture medium at 37 °C and 5% CO_2_, rinsed with PBS, and harvested by trypsin digestion. For the primary cell culture of osteoblasts, bone marrow cells containing osteoclast precursors were isolated from the long bones of 7-month-old female C57BL/6 mice by flushing the marrow from dissected long bones with a syringe and a 25 gauge needle using Hank’s balanced salt solution supplemented with 10% FCS. Erythrocytes were removed by Ficoll-Hypaque density gradient centrifugation and the remaining bone marrow cells were washed with PBS, spun down, and resuspended in culture medium. In 96-well plates, the osteoblasts and bone marrow cells were plated at 1 × 10^4^ cells/well and 2 × 10^5^ cells/well, respectively, in α-MEM supplemented with 10% FCS, antibiotics, and 10 nM 1,25-dihydroxyvitamin D_3_ [14]. At the end of a 6-day culture period, osteoclasts were identified by a TRAP assay.

For primary cell culture of osteoclasts, bone marrow cells containing the desired osteoclast precursors were isolated, added into a sterile 100 mm Petri dish, and cultured in a medium supplemented with 25–50 ng/mL M-CSF. After 48 h, the monolayer was washed with 5 ml ice-cold PBS and the adherent M-CSF-dependent macrophages were gently scraped from the Petri dish. The cells in culture medium were collected and centrifuged at 300 *g* for 3 min. The number of M-CSF-dependent macrophages was then estimated and the cells were then seeded in 96-well plates at 12 × 10^3^ cells per well in 150 ml culture medium supplemented with 25–50 ng/mL M-CSF. M-CSF-dependent macrophages were allowed to attach overnight; the medium was then replaced with culture medium supplemented with M-CSF (25–50 ng/mL) and RANKL (10–120 ng/mL). Fifty percent of this medium was refreshed every 48 h. Osteoclasts began to form after 72–96 h.

### Animal study

Ovariectomized (OVX) mice were subjected to the removal of bilateral ovaries (OVX) under anesthesia with isoflurane (BK Pharm, Goyang, South korea) and sham mice were subjected to incision and suturing without ovary removal (Sham group, *n* = 8). Two weeks after recovering from surgery, OVX mice were randomly divided into the following four groups: OVX mice treated with 0.5% carboxymethyl cellulose (CMC) solution containing 1% sesame oil (OVX group, *n* = 8), OVX mice treated with 80 μg/kg β-estradiol and 800 μg/kg progesterone (E + P group, *n* = 8), and OVX mice treated with 10 or 40 mg/kg EECM (EECM10 or EECM40 group, *n* = 8). The experimental diets were based on the American Institute of Nutrition (AIN)-93 M diets (Dyets Inc., Bethlehem, PA). Samples: EECM or E + P was dissolved in 0.5% CMC solution containing 1% sesame oil. Prepared samples: 0.5% CMC solution containing 1% sesame oil for sham and OVX groups, E_2_ + PGT, or EECM was orally administered to mice. The treatments started 2 weeks after the surgery and lasted 12 weeks. Body weights were measured weekly. Ketamine/xylazine combination solution (K, 90 mg/kg from Huons, Sungnam, South korea; X, 12.5 mg/kg from Bayer korea Ltd., Seoul, South korea) was used by surgical anesthesia in mice. Mice were sacrificed by cervical dislocation under anesthesia and blood samples were collected via abdominal heart puncture for serum isolation. After laparotomy, the uteri and the livers were collected, and the femurs were carefully removed to analyze bone parameters.

### Serum analysis

Serum osteocalcin (OCN) (Quidel, San Diego, CA) and serum estradiol (Cayman Chemical Co., Ann Arbor, MI) were measured using commercial enzyme kits.

### Micro-CT (μCT) scanning

The femoral head was scanned at 0.2 μm intervals to measure the trabecular bone density. The main compressive structure was aligned with the z-axis and the direction of the femur was defined as the x-axis. The upper part of the femur head with the x-axis is the starting point and the neck of the femur is the end of the analysis. Three-dimensional (3D) images of the distal femoral metaphysis were reconstructed by μCT analysis of the growth plate of the right femur distal metaphysis using a SkyScan 1172 (BRUKER microCT Corp., Kontich, Belgium). Tomographic measurements of bone mineral density (BMD), bone mineral content, and bone strength were performed by peripheral quantitative computed tomography using a CTAn (BRUKER microCT Corp.) with a voxel size of 0.2 μm. Image analysis was carried out using integrated SKYSCAN1172 CONTROL software.

### Statistical analysis

Statistical analyses were performed by one-way ANOVA with Dunnett’s multiple comparison test using GraphPad Prism 5 software (GraphPad Software, La Jolla, CA). Differences were considered to be statistically significant at *p* < 0.05.

## Results

### Effects of EECM on osteogenic activity in MC3T3-E1 osteoblast cells through modulation of osteoblastic markers

**Fig. 1:**
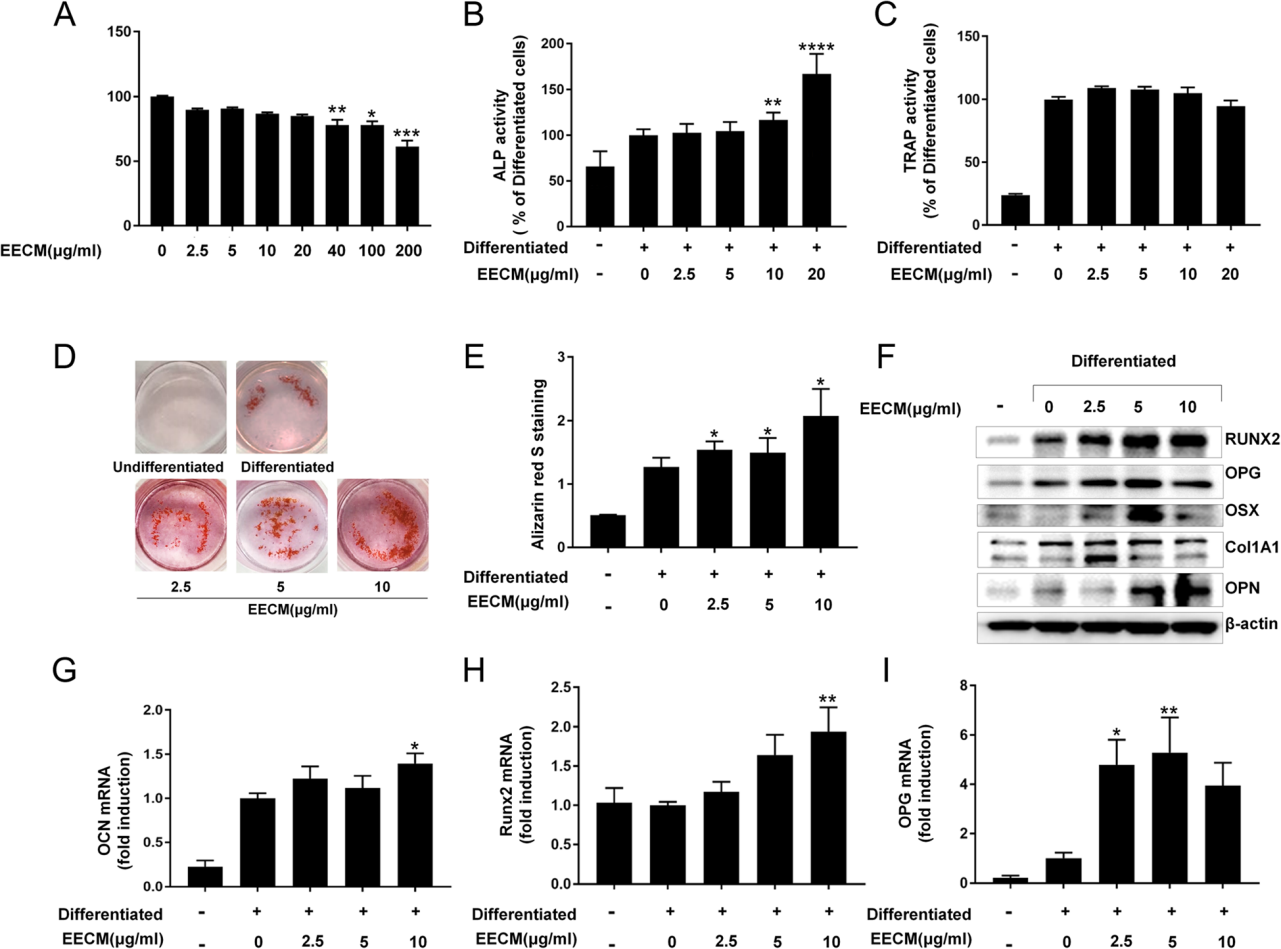
Effect of EECM on osteoblast differentiation of MC3T3-E1 cells and osteoclast differentiation of Raw264.7 cells. **a** Cell viability after 48 h of EECM treatment in MC3T3-E1 osteoblasts. **b** ALP activity in MC3T3-E1 after cells were differentiated for 7 days in differentiation medium with EECM. **c** TRAP activity in RAW264.7 cells after culturing for 7 days with RANKL (50 ng/mL). **d** ARS staining in MC3T3-E1 cells and (**e**) ARS-stained cells. **f** Western blot analysis and (**g-i**) real-time PCR analysis of osteoblastic markers in MC3T3E1 cells. *p < 0.05, **p < 0.01, ***p < 0.001, compared to control and differentiated cells

MC3T3-E1 cells were treated with various concentrations (0–200 μg/mL) of EECM for 48 h to confirm its toxicity. EECM at a concentration of 200 μg/mL was weakly toxic (Fig. 1a). We then investigated whether EECM increased the activity of ALP, a marker of osteoblast differentiation, in MC3T3-E1 cells. Compared with differentiated cells, we found that EECM treatment at 10–20 μg/mL caused significant induction of ALP activity, indicating that EECM induces osteoblast differentiation (Fig. 1b). To investigate the effect of EECM on bone resorption and osteoclasts, we examined the activity of the osteoclast differentiation marker TRAP in RAW 264.7 cells (Fig. 1c). No change in TRAP activity was observed until treatment with 20 μg/mL EECM. The effect of EECM on osteoblast differentiation was confirmed by ARS staining (Fig. 1d and e). EECM significantly induced ARS staining in a dose-dependent manner. Since 2.5-10 μg/ml of EECM were showed sufficient efficiency at ALP activity and Alizarin red S staining, 2.5-10 μg/ml concentrations were used in further studies. We next examined the effects of EECM on osteoblast differentiation markers by western blotting and RT-PCR analysis. EECM increased the Runx2 and OPN protein expression in a dose dependent manner (Fig. 1f). The protein expressions of OPG, OSX and ColA1 were elevated by EECM treatment but 2.5 or 5 μg/mL of EECM was more effective than 10 μg/mL on their expressions. The mRNA expression of Runx2 and OCN was significantly increased in 10 μg/mL of EECM-treated cells compared to differentiated cells, and OPG mRNA expression was significantly elevated in 2.5–5 μg/mL of EECM treated cells (Fig. 1g-i).

### Effects of EECM on bone loss in a model of menopausal osteoporosis in vivo

To determine the therapeutic effect of EECM on menopausal osteoporosis, we used OVX mice as a menopause model. Ovariectomy induced an increase in body weight and a decrease in uterus weight, while E + P treatment allowed maintenance of uterus weight (Table 1).

**Table 1:** Effect of EECM on body weight and uterine weight in vivo. *p < 0.01 and **p < 0.001 versus SHAM group: #*p* < 0.05, ##*p* < 0.01 and ###*p* < 0.001 versus OVX group

Group	SHAM	OVX	E + P	EECM10	EECM40
Body weight (g)					
Initial	17.82 ± 0.47	17.81 ± 0.31	17.82 ± 0.41	17.81 ± 0.40	17.82 ± 0.27
Final	21.79 ± 0.58	24.76 ± 0.38^*^	24.80 ± 1.19^*^	25.34 ± 0.74^***^	25.99 ± 0.86^**^
Difference of body weight (Final-Initial)	3.88 ± 0.49	7.08 ± 0.53^*^	7.05 ± 0.79^*^	8.17 ± 0.43^***^	7.59 ± .071^**^
Uterus (mg)	56.19 ± 4.85	12.59 ± 0.82^**^	24.67 ± 3.44^**,###^	10.46 ± 0.42^**^	11.06 ± 0.46^**^

**Fig. 2:**
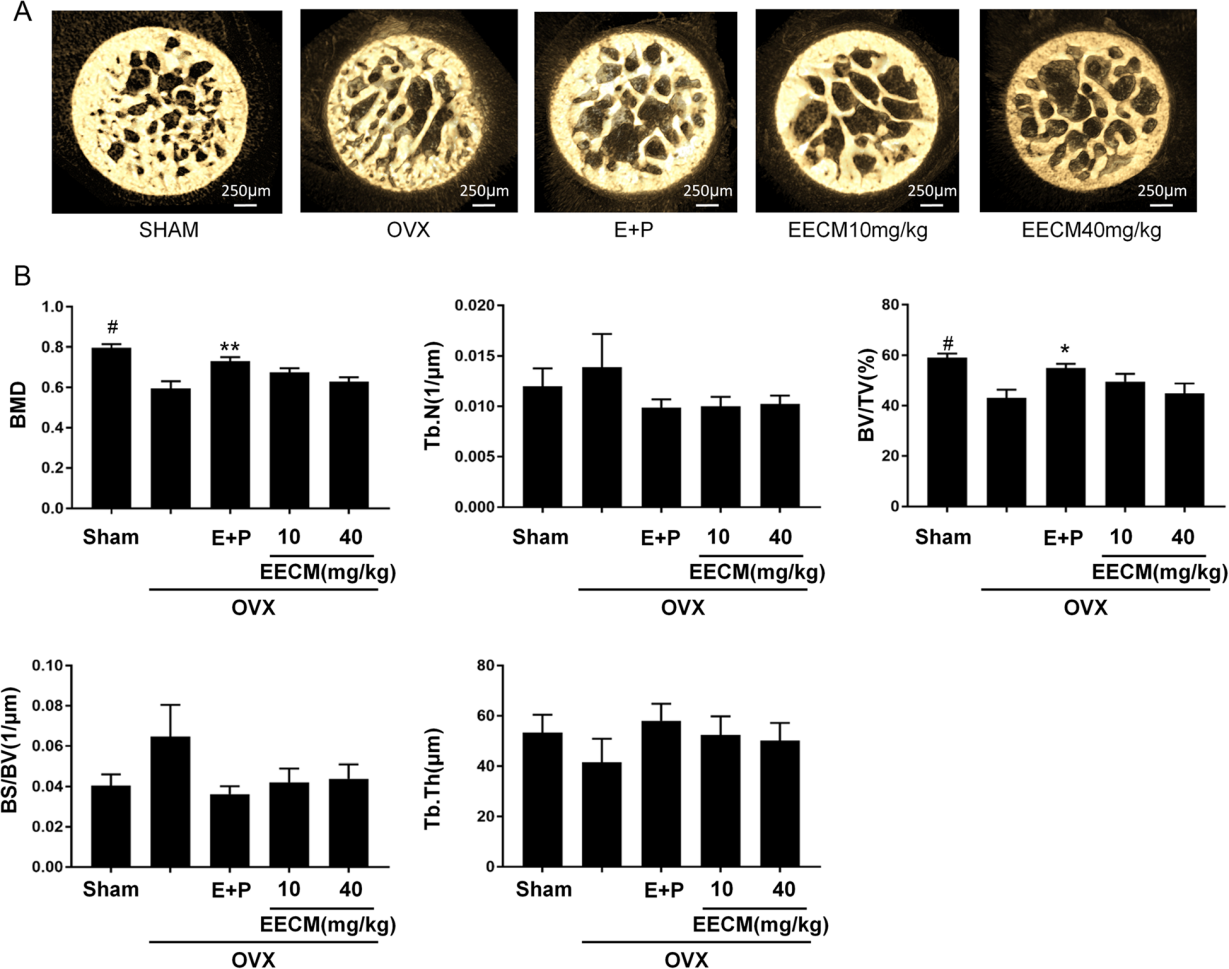
Effect of EECM on bone loss in OVX mice. **a** Morphometric analysis of the right femur distal metaphysis through 3D-μCT and (**b**) tomographic measurements of BMD, BV/TV (%), BS/BV (1/μm), Tb. Th (μm), and Tb. N (1/μm) by computed tomography. #*p* < 0.01 comparing OVX to Sham mice; *p < 0.05 and **p < 0.001 comparing treated to OVX mice

When μ-CT was used to analyze the density of bones in mice, markers of bone density were found to differ between groups (Fig. 2). Pictures of the right femoral head from mice showed that OVX decreased the thickness and size of bone, but E + P and EECM maintained those of bone. The analysis of trabecular bone demonstrated that BV/TV in OVX mice significantly decreased, whereas bone surface to bone volume (BS/BV) and trabecular number (Tb.N) slightly increased compared with that of the Sham group. EECM treatments slightly reversed the bone loss as measured by these parameters; the decreased BMD, BV/TV, and Tb. Th by OVX were increased in the EECM group, whereas increased BS/BV and Tb. N by OVX were decreased. In particular, 10 mg/kg EECM treatment resulted in similar improvements compared with 40 mg/kg. However, the effect of EECM on the bone analysis was not significant.

**Fig. 3:**
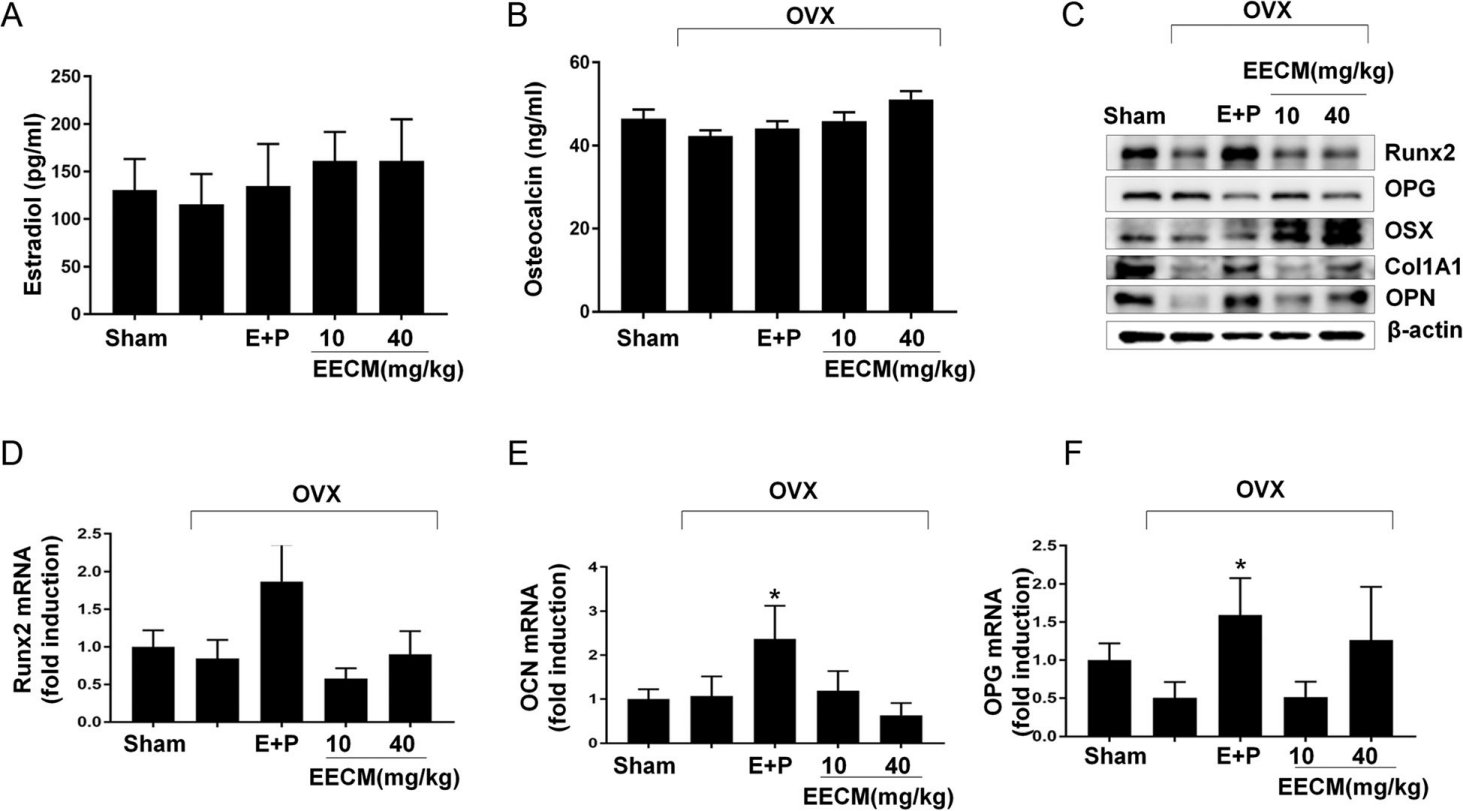
Effect of EECM on serum and osteoblast primary cells from OVX mice. **a** Serum estradiol and (**b**) serum osteocalcin. **c** Western blot analysis and (**d**-**f**) real-time PCR analysis of osteoblastic markers in osteoblast primary cells isolated in mouse model after culturing for 6 days. #*p* < 0.05, comparing OVX to Sham mice; *p < 0.05, **p < 0.01, ***p < 0.001, comparing treated to OVX mice

Although there was no significant differences, serum estradiol was slightly increased in the EECM-treated group compared with the OVX group (Fig. 3a) and serum OCN, a marker of bone mineralization, showed no significant differences in both E + P and EECM-treated groups compared with the OVX group (Fig. 3b). Next, we examined the expression of osteoblast differentiation markers in osteoblast primary cells from the femur of Sham, OVX, and EECM groups by western blotting and RT-PCR analysis. Western blot analysis showed that EECM increased Runx2 and OPG protein expression, and elevated OSX, COL1A1, and OPN in a dose dependent manner (Fig. 3c). In contrast, E + P elevated Runx2, COL1A1, and OPN expression. RT-PCR showed that the mRNA expression of Runx2, OCN and OPG was increased to a greater extent in the E + P groups compared with that in the OVX group but EECM treatment was not critical on the change of those gene expressions (Fig. 3d-f).

### Effects of EECM on osteoblast differentiation and Osteoclastogenesis under co-culture of osteoblasts and osteoclast ex vivo

**Fig. 4:**
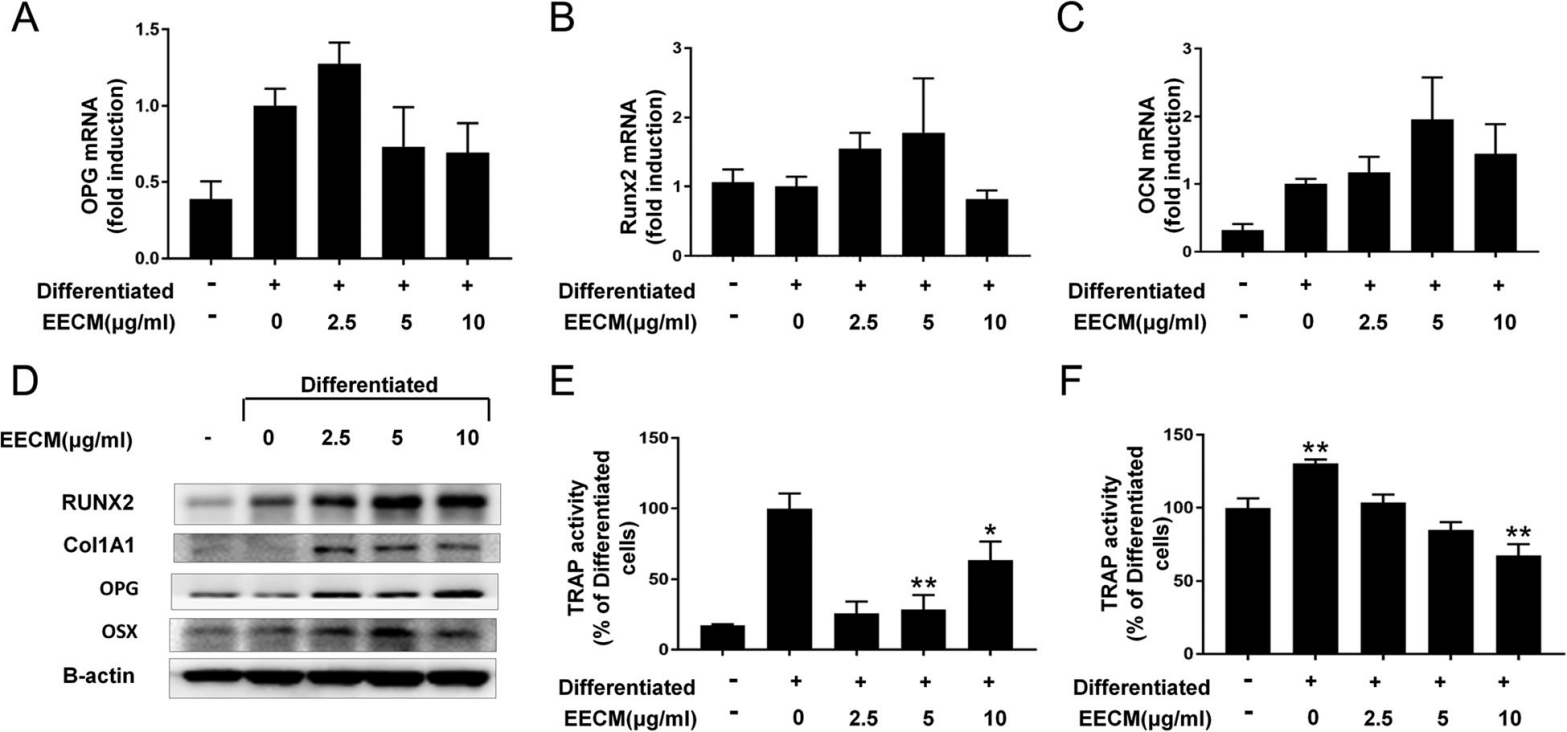
Effect of EECM on mouse primary cells isolated from BL/6 mice. **a-c** Real-time PCR analysis and (**b**) western blot analysis of osteoblastic markers in primary calvarial osteoblasts. TRAP activity in (**e**) mouse primary BMCs and (**f**) co-culture of primary calvarial osteoblasts and BMCs. *p < 0.05, **p < 0.01, ***p < 0.001, compared to differentiated cells

To further confirm the effect of EECM on osteoblastogenesis, we examined the expression of osteoblast differentiation markers in osteoblast primary cells from female BL/6 mice. RT-PCR showed that the mRNA expression of Runx2 and OCN was increased by 2.5–5 μg/mL of EECM treatment, while OPG expression was elevated by 2.5 μg/mL of EECM (Fig. 4a-c). However, there were no significant differences. Western blot analysis showed that EECM increased the protein expression of Runx2, COL1A1, OPG, and OSX (Fig. 4d). To investigate the effect of EECM on bone resorption and osteoclasts, we examined the activity of the osteoclast differentiation marker, TRAP, in M-CSF-induced macrophages. Cells were isolated from female mice bone marrow, cultured for 4 days with M-CSF (50 ng/mL) and with RANKL for differentiation. EECM significantly decreased osteoclastogenesis (Fig. 4e). Isolated BMCs and osteoblasts from the femurs of 7-week-old female mice were co-cultured in the presence of 1α,25-(OH)2D3. We found that EECM decreased the TRAP activity in a dose-dependent manner (Fig. 4f).

### Effects of EECM on osteoblast differentiation via BMP2/4, Smad1/5/9, p38 and estrogen signaling

**Fig. 5:**
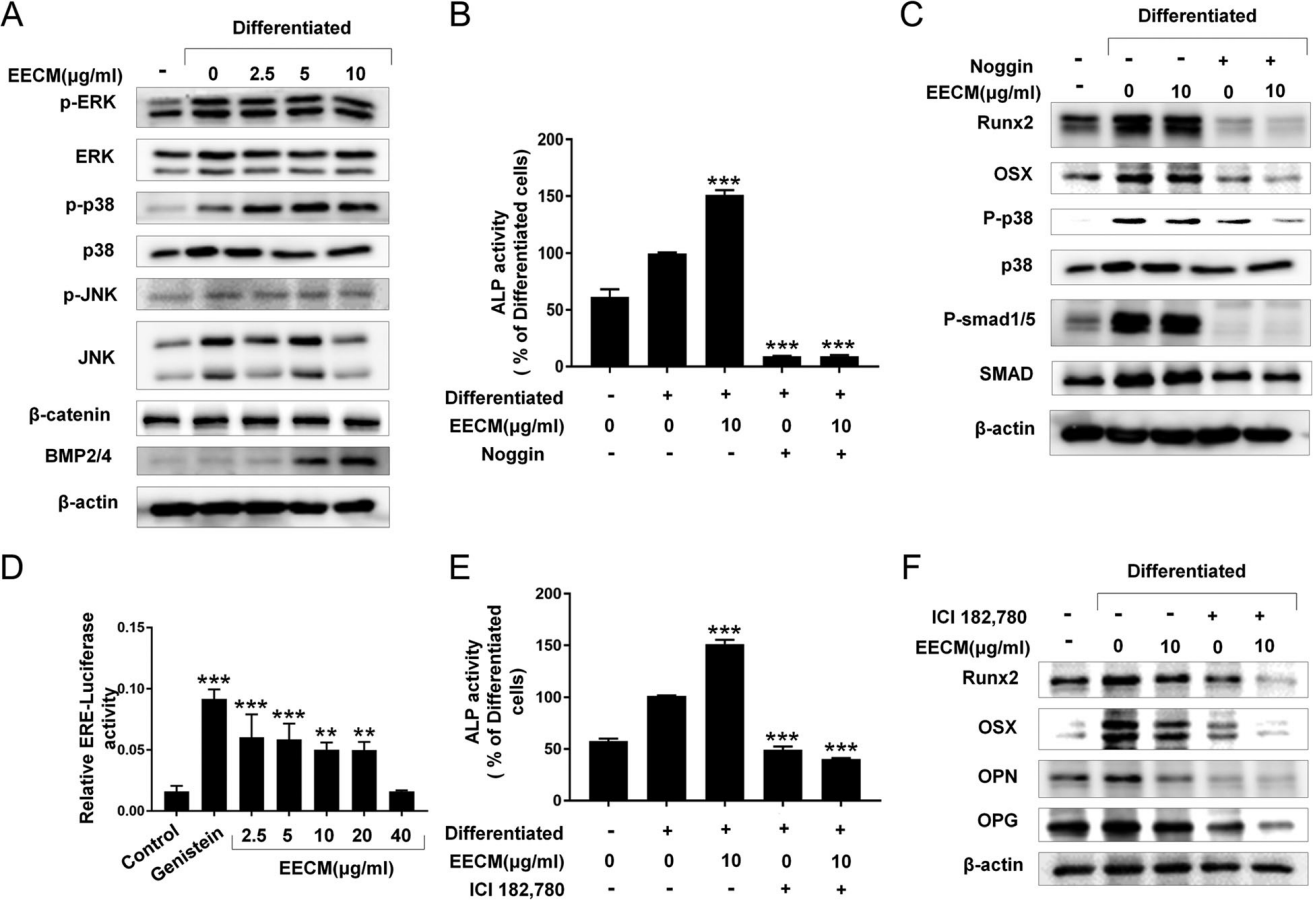
Effect of EECM on BMP-related pathways during osteoblastogenesis. **a** Western blot analysis of MAPK signaling, WNT, and BMP2/4. The effect of the BMP antagonist, Noggin, on (**b**) ALP activity, and (**c**) western blot analysis of osteoblast differentiation markers in MC3T3-E1 cells pretreated with noggin (200 ng/mL) for 1 h, followed by treatment with EECM and retreated after 3 days. **d** Effect of EECM on ERE activity in MCF7 human breast adenocarcinoma cells. Cells were transfected with the ERE and CMV plasmid DNAs and analyzed for luciferase activity after EECM treatment. The effect of the ER antagonist, ICI182.780, on (**e**) ALP activity and (**f**) western blot analysis of osteoblast differentiated markers in MC3T3-E1 cells pretreated with ICI182.780 (10 μM) for 1 h, followed by treatment with EECM and treated after 3 days. *p < 0.05, **p < 0.01, ***p < 0.001, compared to differentiated cells

To further investigate the effects of EECM on osteoblastogenesis, we determined whether EECM alters the upstream signaling of Runx2 and mitogen-activated protein kinases (MAPKs), BMP2/4, and β-catenin. Figure 5a shows that EECM increased the phosphorylation of p38 and BMP2/4, but not that of β-catenin. Because BMP2/4 induced osteogenesis via Smads and p38, we evaluated the role of the BMP antagonist (Noggin) in the effect of EECM. Figure 5b shows that Noggin treatment significantly reduced ALP activity elevated by EECM. Furthermore, phosphorylation of Smad1/5 was simultaneously enhanced in EECM, whereas Noggin blocked EECM-mediated Smad1/5 phosphorylation and Runx2 (Fig. 5c).

To examine the effect of EECM on estrogenic activity, we utilized the ERE-luciferase assay system by transfection of ERE and CMV plasmid DNAs into MCF-7 breast cancer cells. As shown in Fig. 5d, EECM treatment significantly increased luciferase activity by 4–5 times that of the control, which is slightly less than that afforded by treatment with 10 μM of genistein, a representative phytoestrogen. However, 40 μg/mL EECM shows no effect of luciferase activity due to its toxicity (Fig. 1a). ALP activity elevated by EECM was significantly reduced by treatment with the ER antagonist, ICI182,780 (Fig. 5e). The expression of osteoblastogenesis markers, Runx2, BMP-2/4, and p-SMAD1/5/9 was also significantly reduced by treatment with ICI182,780, and the effect of EECM on those markers was decreased by ICI182,780 (Fig. 5f).

## Discussion

Suppression of osteoclastogenesis has been a common strategy for osteoporosis treatment for the past several decades, but recent studies have started to focus more on the use of osteoblastic activators and anabolic agents to restore lost bone mass and/or strength [15–17]. Therefore, in the present study, we investigated EECM as an alternative anti-osteoporotic agent and demonstrated its mechanism of action by focusing on osteogenesis in vitro and in vivo.

First, we found that EECM induces osteoblastogenesis. In our experiments, the significant induction of ALP activity and ARS staining by EECM treatment indicated that EECM has a positive effect on bone restoration because ALP induces the cleavage of polyp-Ca^2+^ resulting in the supplementation of Ca^2+^ to produce hydroxyapatite, a substrate of bone matrix [7]. In addition, EECM treatment elevated BMP2-MAPK-Runx2 signaling, a major signaling pathway in osteoblastogenesis. MAPK and BMP2/4 levels induced by EECM treatment resulted in an increase in Runx2 levels. Runx2 serves as an essential transcription factor, regulating markers involved in the differentiation of mesenchymal cells into preosteoblasts, e.g. OSX, OPN, and COL1A1 [8, 18, 19]. Increased Runx2 induced by EECM resulted in the maturation of osteoblasts due to OSX elevation, mineralization of extracellular matrix due to increase in OCN and OPN, and enhancement of collagen structure due to increase in COL1A1. As a consequence, EECM elevated the amount of mature osteoblasts and enhanced bone density.

Second, we showed that EECM suppresses osteoclastogenesis through osteoblast activation. In our current study, EECM had no effect on TRAP activity in RANK-Ligand induced RAW264.7 cells. However, EECM significantly inhibited TRAP activity ex vivo. Therefore, EECM can directly inhibit the differentiation of bone marrow cells into osteoclasts. In addition, EECM reduced TRAP activity in the osteoblast/osteoclast co-culture system. In the bone remodeling process, OPG constitutes a soluble decoy receptor for RANKL, which is produced by osteoblast/stromal cells and which reduces both the differentiation and function of osteoclasts by blocking the interaction of RANKL with RANK [20]. In our experiments, we found that EECM markedly increased OPG expression without RANKL induction. These results suggested that EECM could also modulate osteoclastogenesis indirectly via OPG secreted from activated osteoblasts.

Third, the effect of EECM on osteoporosis could be mediated by the estrogenic activity of EECM. A previous report showed that EECM has anti-inflammatory activities in rheumatoid arthritis and is rich in phenolic compounds such as luteolin and apigenin [12], which are well known to have estrogenic activities [21, 22]. Our data also confirmed the estrogenic activity of EECM. In addition, the animal model we used, a post-menopausal osteoporosis model induced by ovariectomy, mimics bone disease and bone loss in postmenopausal women as ovariectomy induces estrogen deficiency. As a consequence, imbalance in bone turnover induces fractures due to low bone mass. In this model, EECM treatment reversed the OVX-induced changes in structural parameters. In addition, inhibition of the estrogen receptor compromised the effect of EECM. Estrogen signaling has been known to induce osteoclast apoptosis by activating the Fas ligand. Additionally, ERα is a positive regulator of BMP-2 signaling involved in osteoblast differentiation [23–25]. Collectively, our results strongly suggest that the various phenolic compounds in EECM may acts as estrogenic molecules with beneficial effects on bone mass. Further study is required to shed more light on the identification of active molecules and the estrogenic mechanisms of EECM.

## Conclusion

**Fig. 6:**
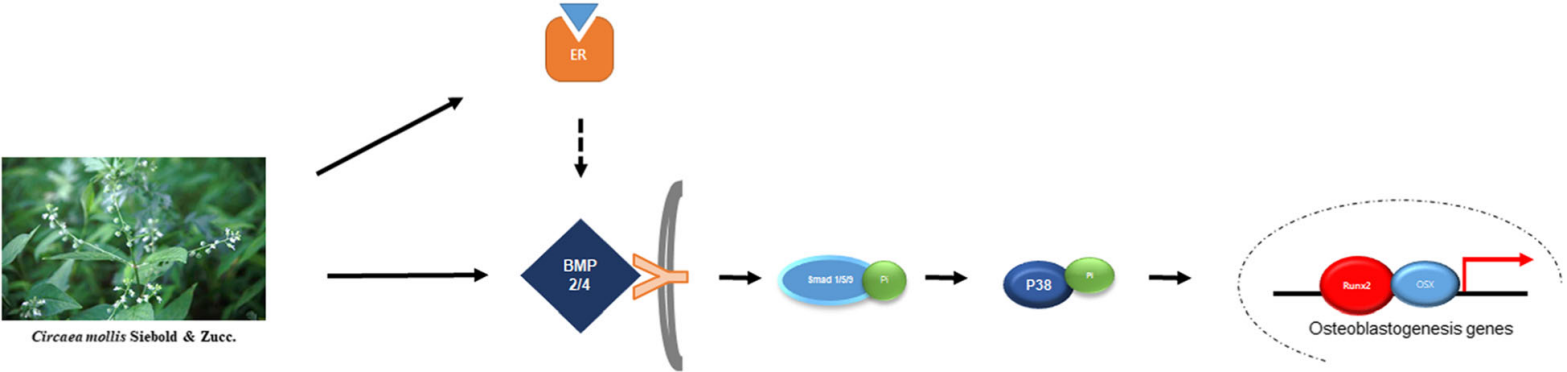
The proposed mechanisms mediating the effects of the EECM on osteoblast differentiation [26]

The present study demonstrated that EECM treatment significantly ameliorates the symptoms of postmenopausal osteoporosis in vivo, by promoting osteoblast differentiation by the induction of BMP2-MAPK-Runx2 signaling, and subsequently by indirect suppression of osteoclastogenesis by OPG. This anti-osteoporotic effect of EECM most likely arose from its estrogenic activity (Fig. 6). Therefore, *Circaea mollis* Siebold & Zucc. may serve as a promising therapeutic reagent to treat postmenopausal osteoporosis.

## Supplementary information

**Additional file 1: Table S1.** Sequences of PCR primers.

**Additional file 2.**

## Data Availability

The data sets used and/or analyzed during the current study available from the corresponding author on reasonable request.

## Abbreviations

ALP: Alkaline phosphatase
α-MEM: alpha minimum essential medium
ARS: Alizarin Red S
BMD: Bone mineral density
BMP-2/4: Bone morphogenetic protein 2/4
BSA: Bovine serum albumin
BV/TV: Bone volume/total volume
FCS: Fetal calf serum
HRT: Hormone replacement therapy
M-CSF: Macrophage colony-stimulating factor
MSC: Mesenchymal stem cell
OCIF: Osteoclastogenesis inhibitory factor
OCN: Osteocalcin
OPG: Osteoprotegerin
OPN: Osteopontin
OSX: Osterix
OVX: Ovariectomized
PBS: Phosphate buffered saline
RANKL: Receptor activator of nuclear factor kappa-Β ligand
RT-PCR: Reverse transcription-polymerase chain reaction
Runx2: Runt-related transcription factor 2
SERM: Selective estrogen receptor modulators
Tb. N: Trabecular number
BS/BV: Bone surface to bone volume
Tb. Th: Trabecular thickness
