# Supplementary material for: Circaea mollis Siebold & Zucc. Alleviates postmenopausal osteoporosis in a mouse model via the BMP-2/4/Runx2 pathway
Source: BMC Complement Med Ther. 2020 Apr 22;20:123. doi: 10.1186/s12906-020-02914-7 (PMC7178630; doi:10.1186/s12906-020-02914-7)
Supplement: Supplementary file 1 — Additional file 1: Table S1. Sequences of PCR primers. [file 12906_2020_2914_MOESM1_ESM.docx]

**Supplementary Table 1. Sequences of PCR primers.**

| Gene | Forward (5’-3’) | Reverse (5’-3’) |
| --- | --- | --- |
| Runx2 | TCCACAAGGACAGAGTCAGATTAC | TGGCTCAGATAGGAGGGGTA |
| Opg | CCGAGGACCACAATGAACA | TCCTGGGTTGTCCATTCAA |
| Ocn | AGACTCCGGCGCTACCTT | CTCGTCACAAGCAGGGTTAAG |
| Gapdh | AAG AGG GAT GCT GCC CTT AC | CCATTTTGTCTACGGGACGA |
