# Supplementary material for: Circaea mollis Siebold & Zucc. Alleviates postmenopausal osteoporosis in a mouse model via the BMP-2/4/Runx2 pathway
Source: BMC Complement Med Ther. 2020 Apr 22;20:123. doi: 10.1186/s12906-020-02914-7 (PMC7178630; doi:10.1186/s12906-020-02914-7)
Supplement: Supplementary file 2 — Additional file 2. [file 12906_2020_2914_MOESM2_ESM.pdf]

Figure1-F

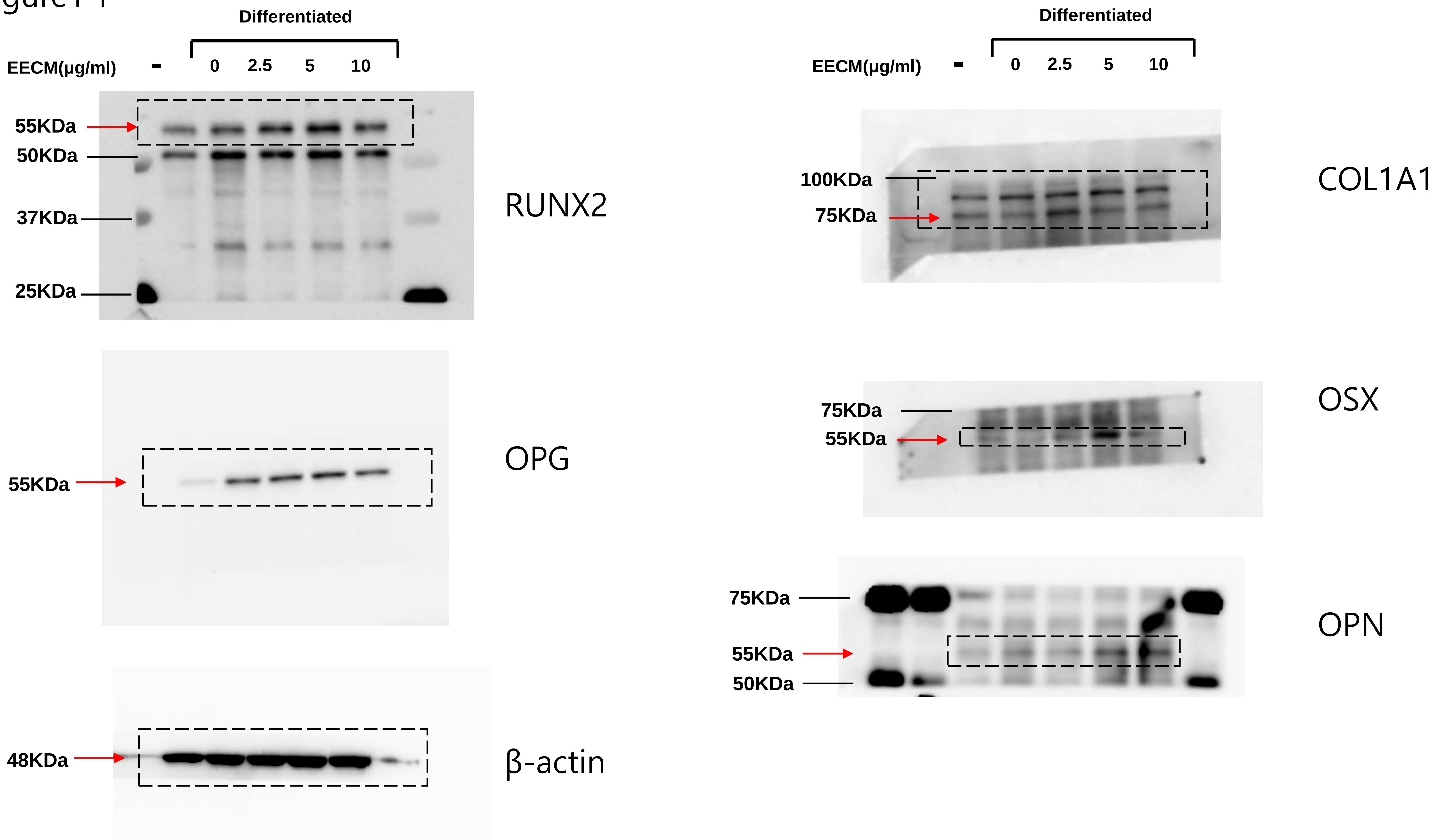

Differentiated
EECM(μg/ml)
-
0
2.5
5
10
55KDa
50KDa
37KDa
25KDa
RUNX2
55KDa
OPG
48KDa
β-actin
Differentiated
EECM(μg/ml)
-
0
2.5
5
10
100KDa
75KDa
COL1A1
75KDa
55KDa
OSX
75KDa
55KDa
50KDa
OPN

Figure3-C

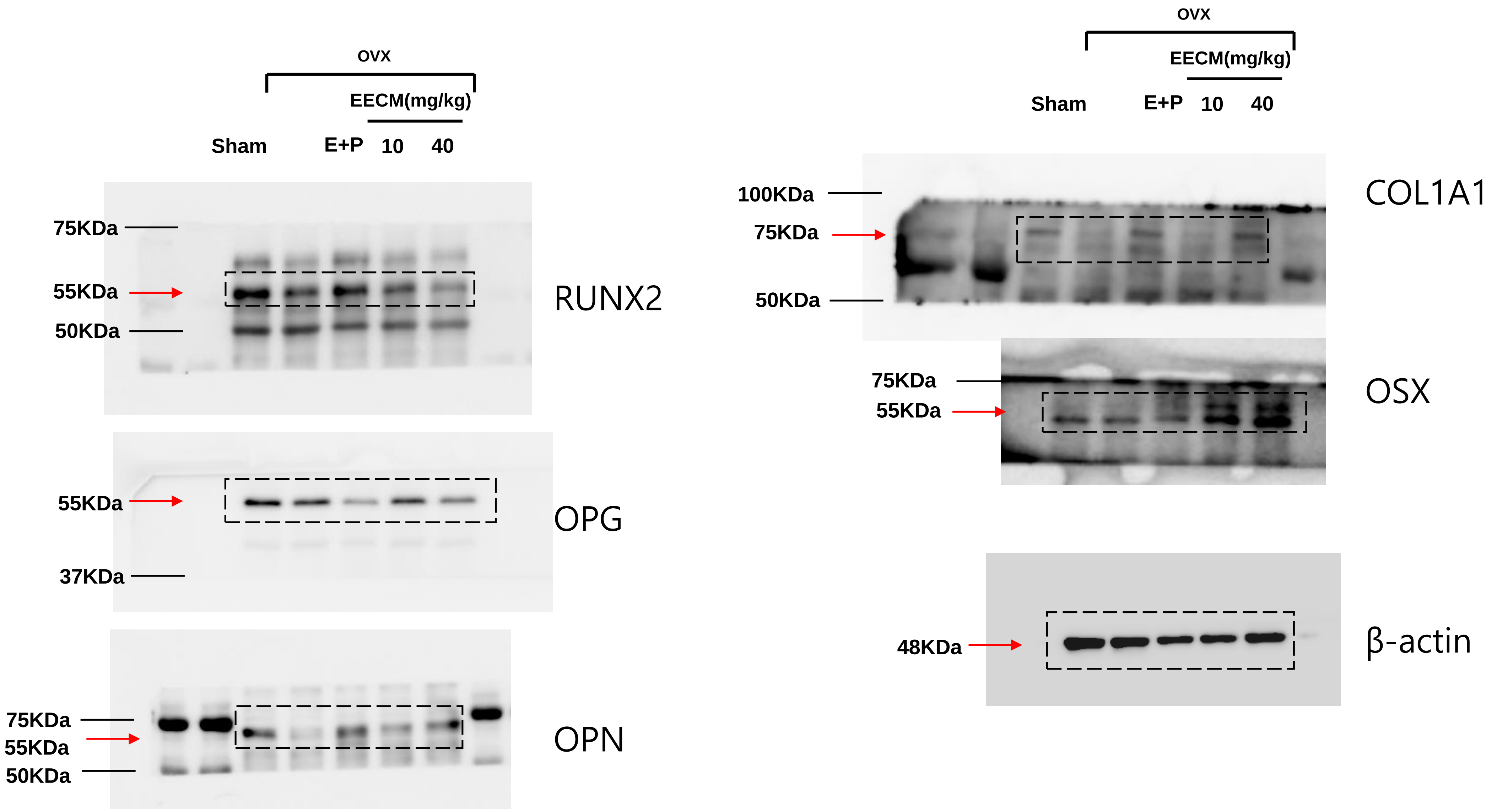
OVX
EECM(mg/kg)
Sham
E+P
10
40
75KDa
55KDa
50KDa
RUNX2
55KDa
37KDa
OPG
75KDa
55KDa
50KDa
OPN
OVX
EECM(mg/kg)
Sham
E+P
10
40
100KDa
75KDa
50KDa
COL1A1
75KDa
55KDa
OSX
48KDa
β-actin

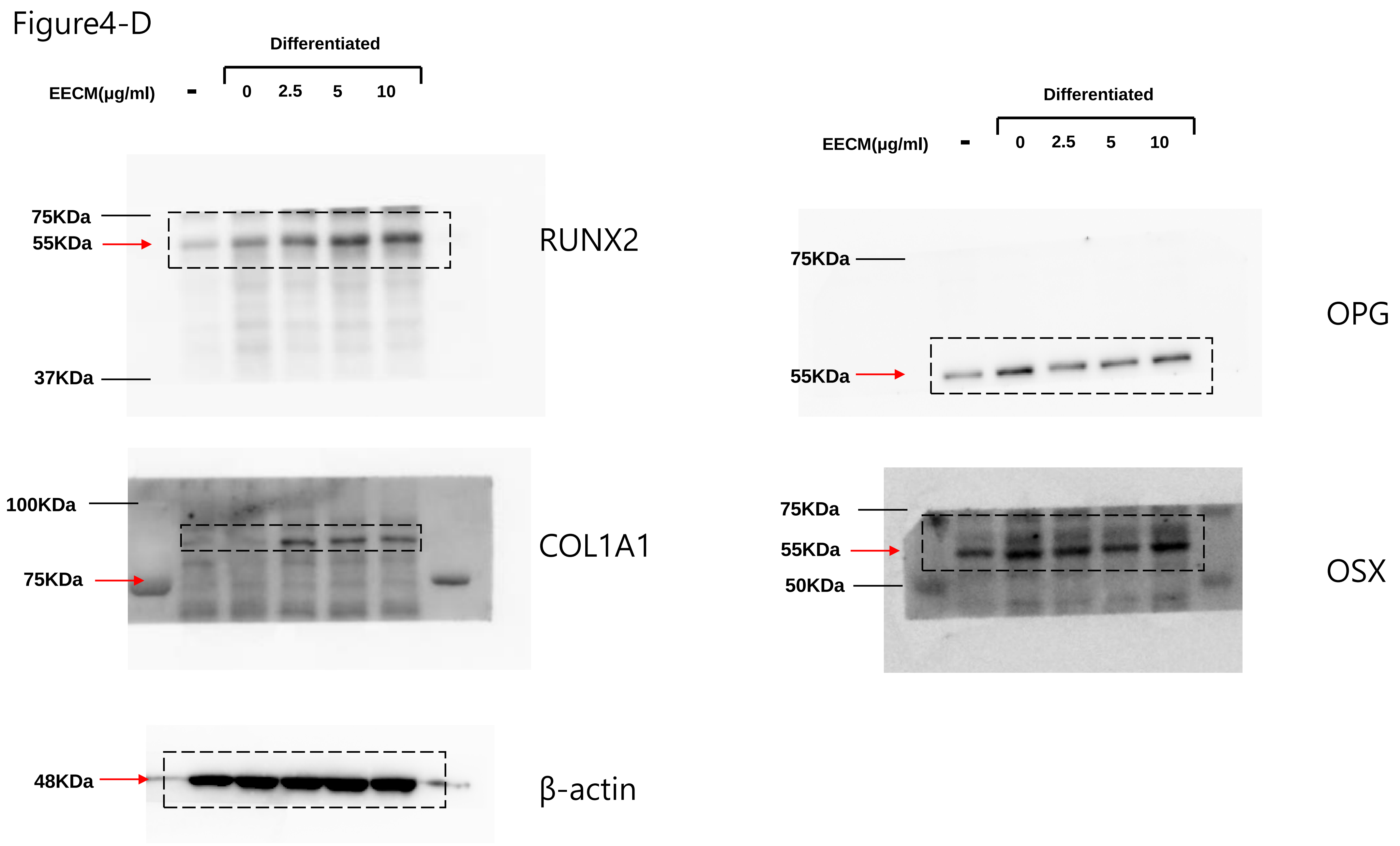

Figure4-D
Differentiated
EECM(μg/ml)
-
0
2.5
5
10
75KDa
55KDa
37KDa
RUNX2
100KDa
75KDa
COL1A1
48KDa
β-actin
Differentiated
EECM(μg/ml)
-
0
2.5
5
10
75KDa
55KDa
OPG
75KDa
55KDa
50KDa
OSX

Figure5-A

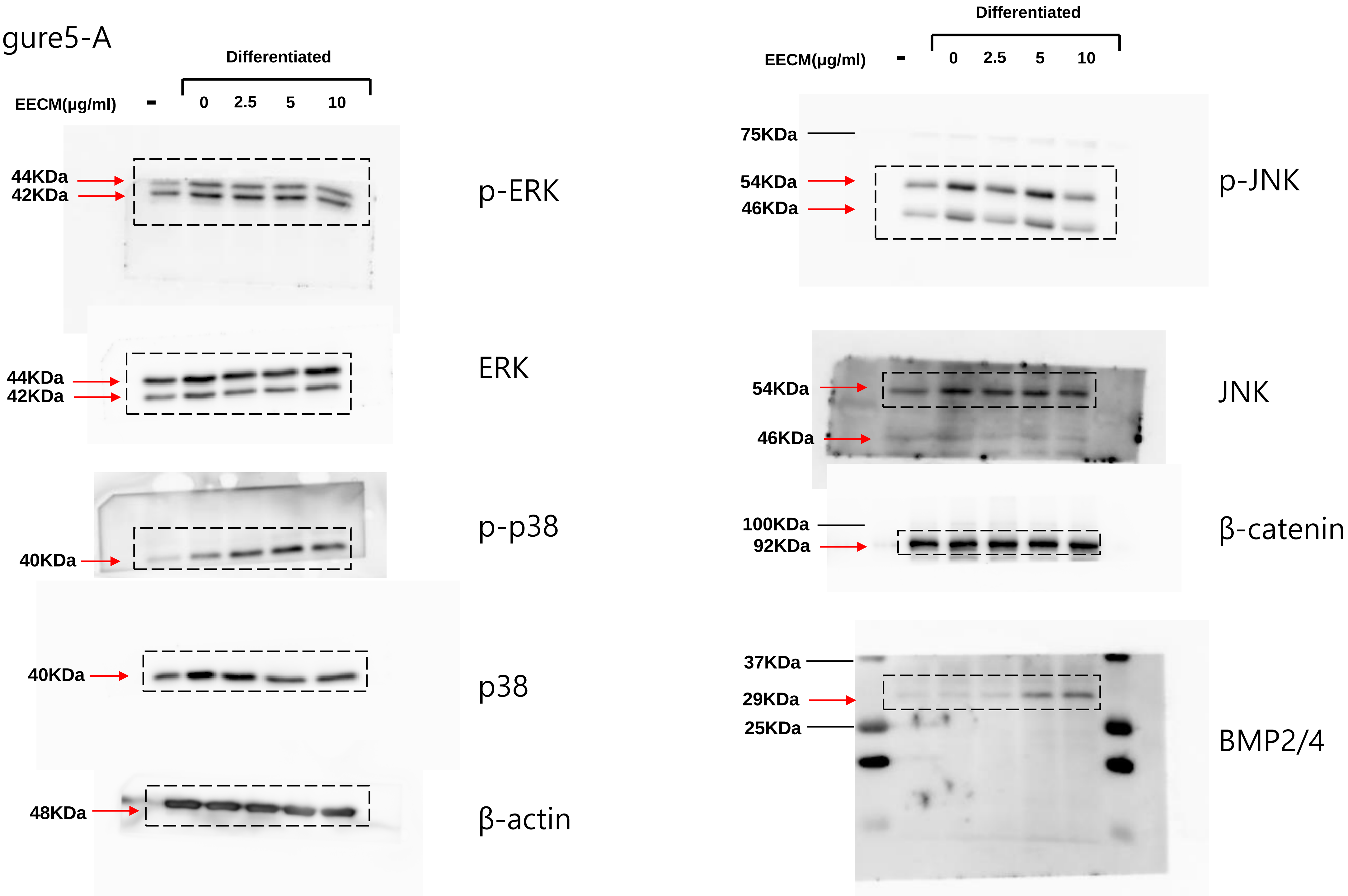

Differentiated
EECM(μg/ml) - 0 2.5 5 10
44KDa
42KDa
p-ERK
44KDa
42KDa
ERK
40KDa
p-p38
40KDa
p38
48KDa
β-actin
Differentiated
EECM(μg/ml) - 0 2.5 5 10
75KDa
54KDa
46KDa
p-JNK
54KDa
46KDa
JNK
100KDa
92KDa
β-catenin
37KDa
29KDa
25KDa
BMP2/4

Figure5-C

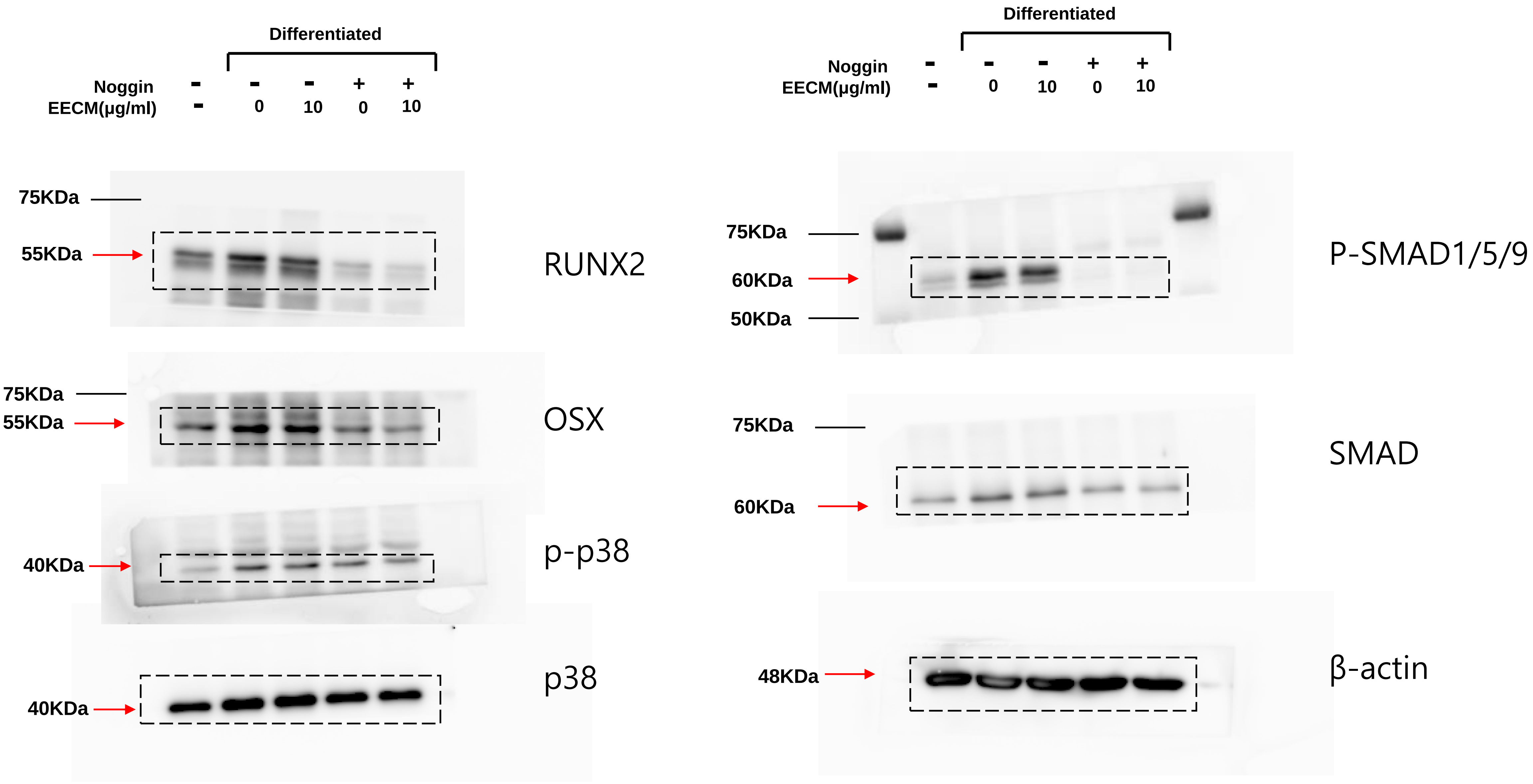

Differentiated
Noggin - - - + +
EECM(μg/ml) - 0 10 0 10
75KDa
55KDa
RUNX2
75KDa
55KDa
OSX
40KDa
p-p38
40KDa
p38
Differentiated
Noggin - - - + +
EECM(μg/ml) - 0 10 0 10
75KDa
60KDa
50KDa
P-SMAD1/5/9
75KDa
60KDa
SMAD
48KDa
β-actin

Figure5-F

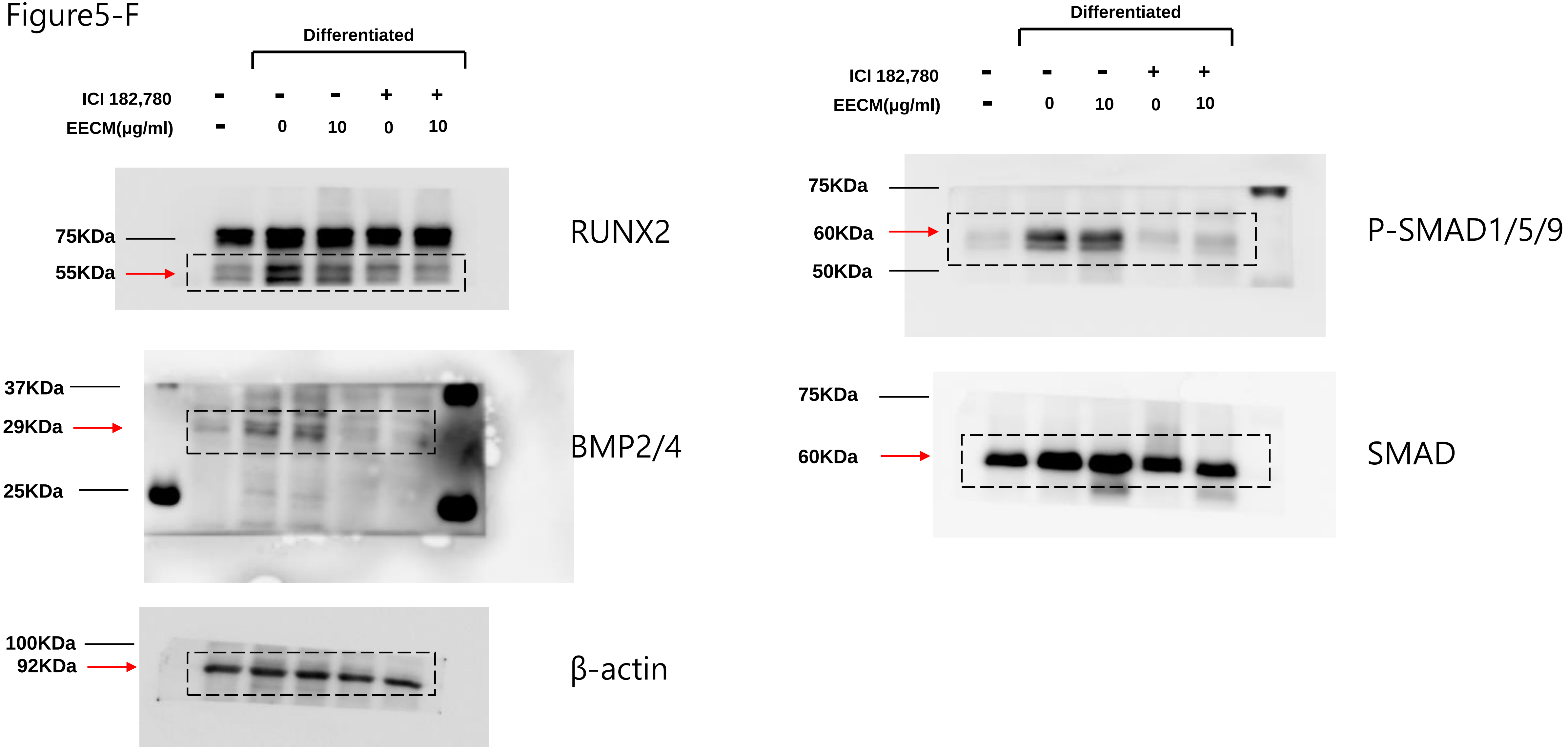

Differentiated
ICI 182,780 - - - + +
EECM(μg/ml) - 0 10 0 10
75KDa
55KDa
RUNX2
37KDa
29KDa
25KDa
BMP2/4
100KDa
92KDa
β-actin
Differentiated
ICI 182,780 - - - + +
EECM(μg/ml) - 0 10 0 10
75KDa
60KDa
50KDa
P-SMAD1/5/9
75KDa
60KDa
SMAD
